# Direct Evidence for a Radial Gradient in Age of the Apple Fruit Cuticle

**DOI:** 10.3389/fpls.2021.730837

**Published:** 2021-10-21

**Authors:** Yiru Si, Bishnu P. Khanal, Oliver K. Schlüter, Moritz Knoche

**Affiliations:** ^1^Fruit Science Section, Institute of Horticultural Production Systems, Leibniz University Hannover, Hannover, Germany; ^2^Department of Horticultural Engineering, Leibniz Institute for Agricultural Engineering and Bioeconomy (ATB), Potsdam, Germany

**Keywords:** *Malus* × *domestica*, cuticle, cutin, wax, strain, stress

## Abstract

The pattern of cuticle deposition plays an important role in managing strain buildup in fruit cuticles. Cuticular strain is the primary trigger for numerous fruit-surface disorders in many fruit crop species. Recent evidence indicates a strain gradient may exist within the apple fruit cuticle. The outer layers of the cuticle are more strained and thus more susceptible to microcracking than the inner layers. A radial gradient in cuticle age is the most likely explanation. Our study aimed to establish whether (or not) deposition of new cutin in a developing apple fruit occurs on the inner surface of the cuticle, i.e., immediately abutting the outward-facing epidermal cell wall. Developing apples were fed with ^13^C oleic acid through the skin. Following a 14-d period for incorporation, the fruit was harvested and the cuticular membranes (CMs) isolated enzymatically. The CMs were then ablated to varying extents from the inner or the outer surfaces, using a cold atmospheric pressure plasma (CAPP). Afterwards, the ablated CMs were dewaxed and the ^13^C contents were determined by mass spectrometry. The incorporation of ^13^C in the cutin fraction was higher than in the wax fraction. The ^13^C content was highest in non-ablated, dewaxed CM (DCM) and decreased as ablation depth from the inner surface increased. There was no change in ^13^C content when ablation was carried out from the outer surface. As fruit development proceeded, more ^13^C label was found towards the middle of the DCM. These results offered direct evidence for deposition of cutin being on the inner surface of the cuticle, resulting in a radial gradient in cuticular age—the most recent deposition (youngest) being on the inner cuticle surface (abutting the epidermal cell wall) and the earliest deposition (oldest) being on the outer surface (abutting the atmosphere).

## Introduction

A cuticular membrane covers the outside of all primary-skin surfaces of all organs of terrestrial plants, specifically all leaves, many stems, and fruit. The cuticular membrane (CM) is a non-living extracellular polymer, deposited on the outer surface of the cell walls of the epidermis. It comprises an insoluble polymer matrix “cutin,” solvent-soluble lipids “waxes,” and cell wall polysaccharides (Schreiber and Schönherr, 1990; Dominguez et al., 2011; Yeats and Rose, 2013). The waxes are deposited within the CM as intracuticular waxes and also on the CM surface as epicuticular waxes. The primary function of the cuticle is to present a barrier against uncontrolled exchanges of respiratory gases (Jeffree, 2006) and water (Riederer and Schreiber, 2001; Kerstiens, 2006) and invasion by pathogens (Huang, 2001; Heredia, 2003; Serrano et al., 2014). To continue its barrier functions, the cuticle must maintain its functional integrity throughout the life of a leaf or a fruit.

Maintenance of functional integrity presents a particular challenge to the cuticles of fruit. In contrast to leaves, a fruit skin is subject to an extended period of strain, as a fruit surface usually continues to extend from flowering through to fruit maturity, commonly a period of about five months. The epidermal and hypodermal cells beneath the cuticle cope with these growth strains by a combination of cell division, cell expansion, and, in some fruit skins, by a change in epidermal cell aspect ratio (Maguire, 1998; Knoche and Lang, 2017). However, the polymeric CM cannot grow or divide but is simply stretched out by the ongoing area growth of the underlying epidermis (Knoche et al., 2004). This ongoing strain can result in the formation of cuticular microcracks that compromise its barrier functions. Microcracking is aggravated by surface wetness (Khanal et al., 2021). Moreover, microcracks are the first visible symptoms of several important fruit-surface disorders, including russet, skin spots, neck shrivel, and macrocracks (Skene, 1980; Knoche and Lang, 2017). Throughout fruit development, the cuticle of an apple copes with the ongoing strain by the ongoing deposition of new cutin and wax (Lai et al., 2016)—else the stretched CM would become thinner and thinner. The continuing addition of new cutin to the extending cuticle and its impregnation with intracuticular waxes “fix” the strain, converting the elastic strain component into a plastic component (Khanal et al., 2013a).

Previous studies have shown that these processes result in the development of a radial gradient of strain across the CM, with the material on the inner side (abutting the cell walls) being less strained and that on the outer side (abutting the atmosphere) being more strained (Khanal et al., 2014). In this way, it is most common for a microcrack to appear first on the outer side of the CM and for this crack gradually to propagate deeper into the cuticle as straining continues, so as eventually to traverse the CM through to the inner (cell) side (Knoche et al., 2018). The most likely explanation for the observed radial gradient in strain is a corresponding gradient in the deposition and thus the age of the cuticle. Additional factors that may contribute to a radial gradient in strain are the presence of polysaccharides on the inner side of the cuticle (Dominguez et al., 2011), a compositional gradient of C16/C18 cutin monomers within the cuticle with C18 fatty acids having a higher impact on cuticle integrity (Kolattukudy and Walton, 1972; Walton and Kolattukudy, 1972; Kolattukudy et al., 1974; Kolattukudy, 1980; Straube et al., 2021) and/or changing status of cutin polymerization (Espana et al., 2014; Martin and Rose, 2014).

It was hypothesised that with cutin being added preferentially to the inner side of the CM, this region will be younger, and so it will have suffered a shorter history of expansion, and so it will be less strained than the outer side. Taking the opposite view, the cutin on the outer side of the CM will have been deposited earlier on in the life of the fruit, and so be older, and so have suffered a longer history of expansion, and so be more strained. This hypothesis would explain why microcracking usually begins on the outer side of the CM. It would also explain why dewaxed CMs (DCM) usually “curl” following extraction of wax. Unfortunately, direct evidence for the deposition and age gradients in the cuticle is lacking, i.e., that deposition occurs on the inner surface of the cuticle.

Therefore, the objective of this study was to provide direct evidence for a radial gradient in cuticle deposition and age. We first fed ^13^C labelled oleic acid to the fruit surface. This was incorporated into the CM (Si et al., 2021a,b). Following feeding and incorporation, the cuticle was enzymatically isolated and then ablated from its inner surface, or its outer surface, using a cold atmospheric pressure plasma (CAPP). Thereafter, the ^13^C content of the ablated CM was determined. We focused on the cutin fraction, since an association of ^13^C with the wax fraction may have simply resulted from partitioning (Si et al., 2021a). We chose the ‘Idared’ apple for our study because ‘Idared’ is a russet non-susceptible cultivar where surface wetness during feeding does not trigger russet formation (Khanal et al., 2013b, 2021; Chen et al., 2020).

## Materials and Methods

### Plant Material

‘Idared’ apple (*Malus* × *domestica* Borkh.) trees grafted on M9 rootstocks were cultivated in the Horticultural Research Station of the Leibniz University Hannover at Ruthe, Germany (lat. 52°14′N, long. 9°49′E) according to current EU regulations for integrated fruit production. Representative fruits of normal growth and free from visible blemishes were selected for the experiments.

### Methods

#### Fruit Growth and Cuticle Deposition

Fruits were harvested at different stages of development and the mass of each was recorded. The surface area was calculated assuming sphericity and a mean density of 1 kg dm^−3^. A sigmoid regression line was fitted through plots of fruit surface area vs. time in days after full bloom (DAFB) and fruit mass vs. DAFB. The number of replicates at each time was 60.

Cutin and wax deposition was quantified using enzymatically isolated CM using standard procedures. Briefly, epidermal skin segments (ES) were excised from the equatorial plane of fruit using a biopsy punch (8 mm, Acuderm Inc., FL, USA). The ES was incubated at ambient laboratory temperature in an isolation medium containing pectinase (90 ml L^−1^, Panzym Super E flüssig, Novozymes A/S, Krogshoejvej, Bagsvaerd, Denmark) and cellulase (5 mL L^−1^, Cellubrix L, Novozymes A/S). The enzyme solution was buffered in 50 mM citric acid and the pH adjusted to 4 using sodium hydroxide (NaOH). To avoid microbial growth, sodium azide (NaN_3_) was added at a final concentration of 30 mM. The enzyme solution was periodically refreshed until the CMs separated from the underlying tissue. The CMs were cleaned using a soft camel-hair brush and thoroughly rinsed with deionized water.

To determine the mass per unit area, CMs were dried overnight at 40°C and then weighed on a microbalance (CPA2P; Sartorius, Göttingen, Germany). Subsequently, the CMs were Soxhlet extracted using a chloroform:methanol mixture (1:1 v/v) for 2.5 h. Dewaxed CMs (DCM) were again dried overnight and weighed.

#### Dosing Procedure and Cuticle Preparation

##### Feeding ^13^C Labelled Oleic Acid

The solutions were prepared by dissolving uniformly ^13^C labelled oleic acid (> 95% purity, Larodan AB, Solna, Sweden) in 0.05% surfactant solution (Glucopon 215 UP/Mb; BASF, Ludwigshafen, Germany) at a final concentration of 167 μM (equiv. to 50 mg L^−1^). Solutions were vortexed for at least 3 min immediately after preparation and again for 3 min immediately before application to the fruit surface. Donor solutions were always prepared fresh on the day of use.

**Figure 1:**
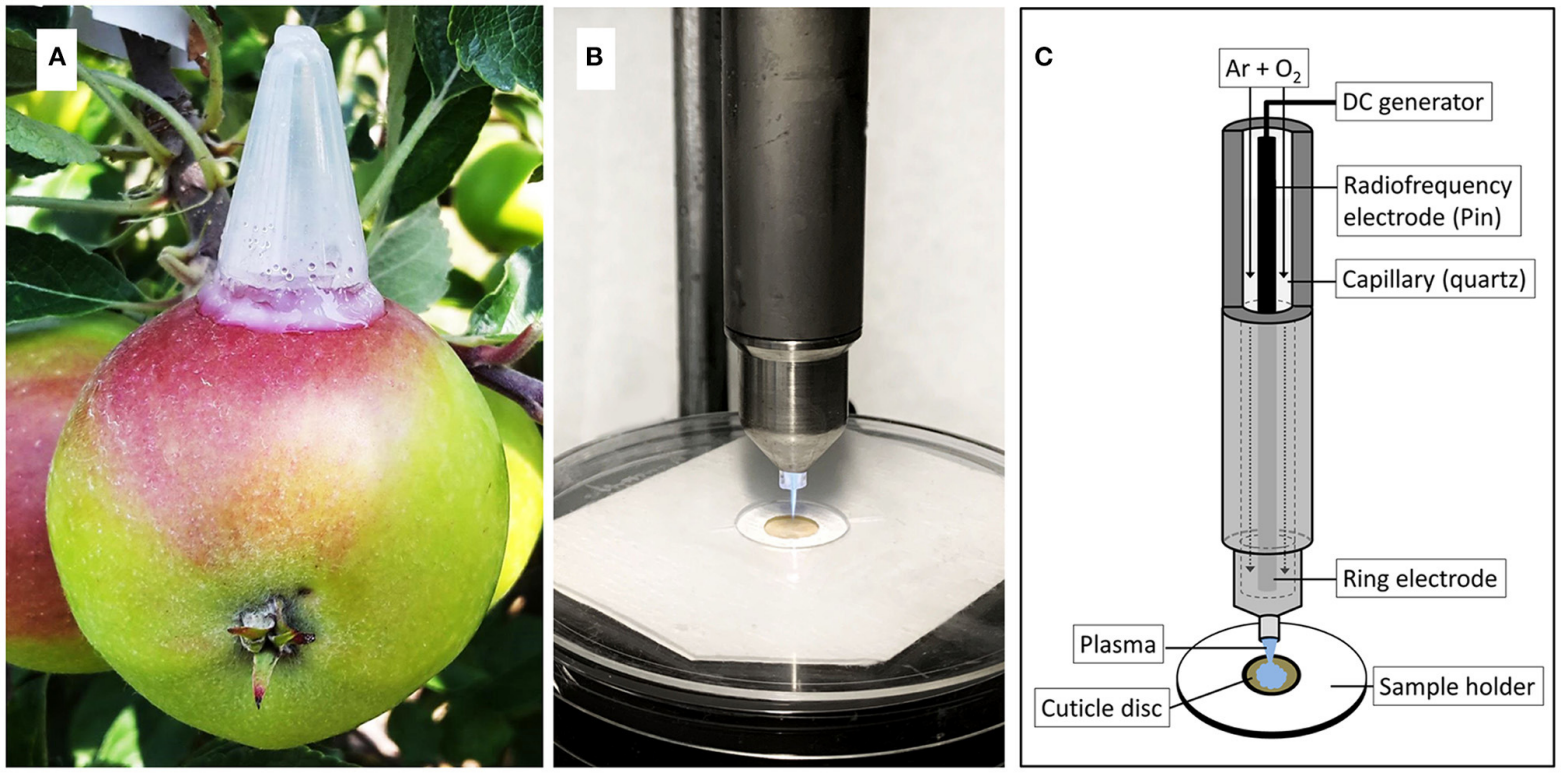
Apple fruit with mounted polyethylene tube for feeding ^13^C labelled oleic acid under field conditions **(A)**. Cold atmospheric pressure plasma (CAPP) is generated by a plasma jet during ablation of the cuticular membrane (CM) **(B)**. Sketch of plasma jet and CM sample holder illustrating the various parts of the plasma jet and the experimental setup **(C)**. The sketch was adapted from Bußler (2017).

The solution was applied as described earlier (Si et al., 2021a,b). Briefly, polyethylene tubes (25 mm height, 14 mm diameter) with a tapered tip and a minute hole in the tip were mounted in the equatorial region of the apple fruit using a non-phytotoxic silicon rubber (SE 9186 RTV; Dow Toray, Tokyo, Japan). A volume of 400 μL of donor solution was injected through the hole in the tip of the tube, and the hole was sealed using silicone rubber to prevent drying of the donor solution (Figure 1A). Feeding was terminated after 7 d when the tubes were removed. The original footprint of the tube was then marked with a permanent marker and the marked area was rinsed with deionized water. Fruits were sampled either 14 d after the termination of feeding or at commercial maturity.

##### Cuticle Isolation

After harvest, the marked area of the fruit surface was rinsed with 1% surfactant solution (Glucopon 215 UP/Mb; BASF) and blotted dry. A 12 mm diameter ES was excised from the central region of the marked area using a biopsy punch (Acuderm Inc., FL, USA). The CMs were isolated from the ES as described above. Isolated and cleaned CMs were stored in deionized water at ambient temperature until use.

#### Cold Atmospheric Pressure Plasma (CAPP) Treatment

The CMs were dried overnight at 40°C and weighed on a microbalance. The CM discs were mounted between two discs of thick paper. The upper paper disc had an 8 mm diameter hole in the centre. The paper/CM/paper “sandwich” was then positioned on a custom-made sample holder such that the CM surface was exposed to the plasma jet (Figures 1B,C). This setup prevented any movement of the CM during exposure to CAPP. The CAPP was generated from a mixture of 99.9% argon and 0.1 % oxygen (Air Liquide, Düsseldorf, Germany) using an 8 W plasma jet (kINPen 09; Neoplas tools, Greifswald, Germany) at ambient temperature and pressure (Weltmann et al., 2009). When the mixture of gases passed through the electrode operated at a high-frequency voltage (1.1 MHz; 2–6 kV peak-to-peak voltage), the CAPP was generated at the tip of the electrode (Figures 1B,C). The flow rate of the gas mixture was set at 5.4 L min^−1^ (Multigas controller, 647C; MKS Instruments, Andover, MA, USA). The power supplied to the plasma jet was 65 V at a resonance balancing of 0.05 A.

The CM discs mounted on the sample holder were subjected to CAPP treatment of the inner or outer side for durations of 0-, 5-, 10-, 15-, 20-, or 25-min. Earlier studies established that increasing exposure times to CAPP results in increasing ablation of synthetic polymers (Clouet and Shi, 1992) and isolated cuticles irrespective of the orientation of the cuticle (Khanal et al., 2014).

The distance between the CM and the tip of the plasma jet was set to 8 mm. This setup produced a CAPP treated area of about 8 mm diameter in the centre of the CM disc. Using these settings, the temperature of the CM disc always remained below 40°C (Khanal et al., 2014).

#### Scanning Electron Microscopy (SEM)

The effect of CAPP treatment on the outer and inner surfaces of the CMs was established using SEM. Non-ablated control CMs and CAPP treated CMs following wax extraction were observed in a Quanta 200 SEM (FEI Europe Main Office, Eindhoven, The Netherlands). Cross-sections were viewed following freeze fracturing in liquid nitrogen. Specimens were mounted on aluminium stubs using conducting carbon tape and sputter-coated with gold. Calibrated images of the inner and outer surfaces were prepared at 1,000 x, those of cross-sections at 500 x. The acceleration potential was 15 kV.

#### Measurement of CM and DCM Mass

The mass loss during ablation of the CM by the CAPP treatment was quantified on a core disc excised from the ablated CMs. The core disc was of 4 mm diameter and was excised using a biopsy punch. The CMs were dried overnight at 40°C and weighed on a microbalance. The mass per unit area was calculated. Thereafter, the CMs were extracted in 2.0 ml chloroform:methanol (1:1, v/v) per disc for 24 h at ambient temperature. The dewaxed CMs were removed from the chloroform:methanol extraction mixture, rinsed once with fresh 0.5 ml chloroform:methanol, then dried overnight at 40°C. The DCM discs were then weighed and their mass per unit area calculated.

#### ^13^C Quantification Using Isotope Ratio Mass Spectrometry (IRMS)

The amount of unlabelled (^12^C) and labelled carbon (^13^C) in the 4 mm diameter CMs and DCMs (after CAPP treatment of the CMs) were measured on an elemental analyser (Isotope Cube; Elementar, Hanau, Germany) coupled with an isotope ratio mass spectrometer (Isoprime precisION; Isoprime-Elementar, Manchester, UK). We followed the procedure used by Si et al. (2021a,b). The labelled CM and DCM discs were crimped in aluminium boats (one disc per boat) (6 × 6 × 12 mm; LabNeed GmbH, Nidderau, Germany). The samples were combusted at 1,080°C under a pulse of oxygen. Cerium dioxide was supplied to catalyse the combustion. The resulting CO_2_ was passed to an isotope ratio mass spectrometer where the standard and isotopic C contents were quantified by a heat conductivity detector. For each measurement, the detector was calibrated using a commercial sediment standard.

The C isotope ratio was calibrated online by injecting one pulse of the reference gas. The isotopic composition of C was calculated in the delta notation (at%) and referenced against Vienna Pee Dee Belemnite (VPDB). Further, C (at%) was referenced using international standards supplied by the International Atomic Energy Agency (IAEA, Vienna, Austria).

Sucrose (IAEA-CH-6), cellulose (IAEA-CH-3), and caffeine (IAEA-600) were used as standards for isotopic composition, and an in-house standard made from the spruce litter was used as an internal standard for quality control of C composition and the referenced isotopic composition.

The relative amount of tracer-derived C (*R*_*Tracer*_) (new carbon) to the total carbon pool (old plus new carbon) was calculated using equation (1) (Gearing, 1991).


(1) $$\begin{array}{l} R_{Tracer}=\;\frac{at\%\;L-at\%\;C}{at\%\;C-at\%\;T}\times 100 \end{array}$$

In this equation, at% represents the at% value of tracer (T) and labelled (L) or unlabelled control (C) CM or DCM sample. The total mass of tracer in the whole CM or DCM sample (*M*_*Tracer*_) was calculated using equation (2).


(2) $$\begin{array}{l} M_{Tracer}=\;\frac{R_{Tracer}\times \;M_{Sample}\times \;\%C}{m_{sample}} \end{array}$$

where *M*_*Sample*_ represents the total mass of the 4 mm diameter CM or DCM disc combusted in the elemental analyser, %C represents the carbon content of the respective sample, and *m*_*sample*_ represents the molar mass of carbon in the sample. All % values used in the above equations were divided by 100 prior to calculation.

### Data Analyses

All experiments were conducted and analysed using completely randomised designs. Data were analysed by linear regression analysis using the statistical software package SAS version 9.1.3 (SAS Institute, Cary, NC). Data are presented as means ± standard errors. Where not shown, the error bars were smaller than data symbols.

## Results

**Figure 2:**
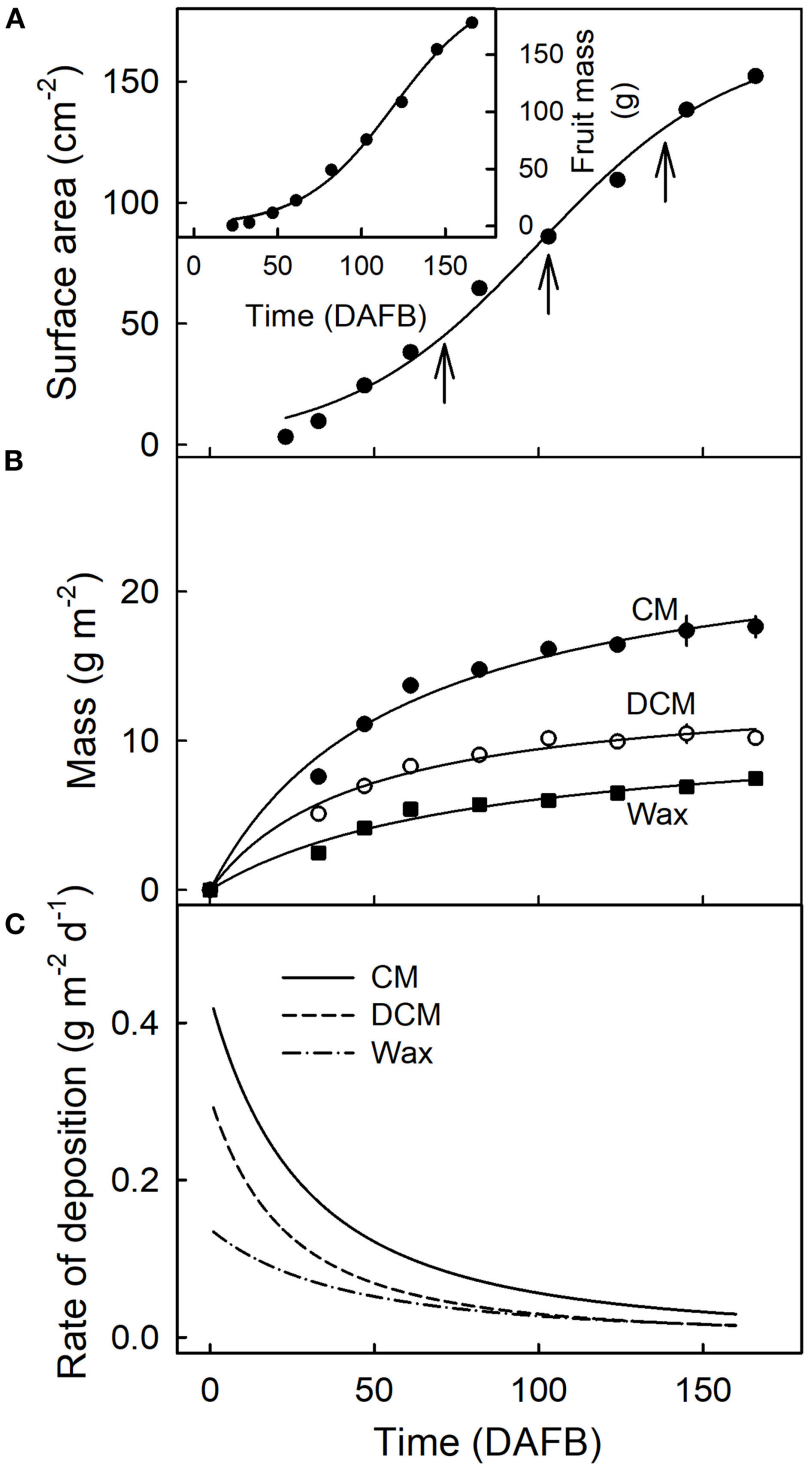
Fruit surface area **(A)**, fruit mass [**(A)**, inset]and mass per unit area of the cuticular membrane (CM), the dewaxed CM, and the wax **(B)** during the development of apple fruit. **(C)** Rates of deposition of CM, DCM, and wax. The x-axis scale is in days after full bloom. The vertical arrows in A indicate the beginning of the feeding periods of ^13^C oleic acid to the outer surface of developing apple fruit. Data points represent means ± SE, *n* = 15–60.

Fruit surface area and fruit mass increased sigmoidally with time (Figure 2A). The masses of CM, DCM, and wax per unit fruit surface area, all increased during fruit development (Figure 2B). The rates of deposition of cutin and wax were highest in the early stages of fruit development, decreasing steadily until maturity (Figures 2B,C).

**Figure 3:**
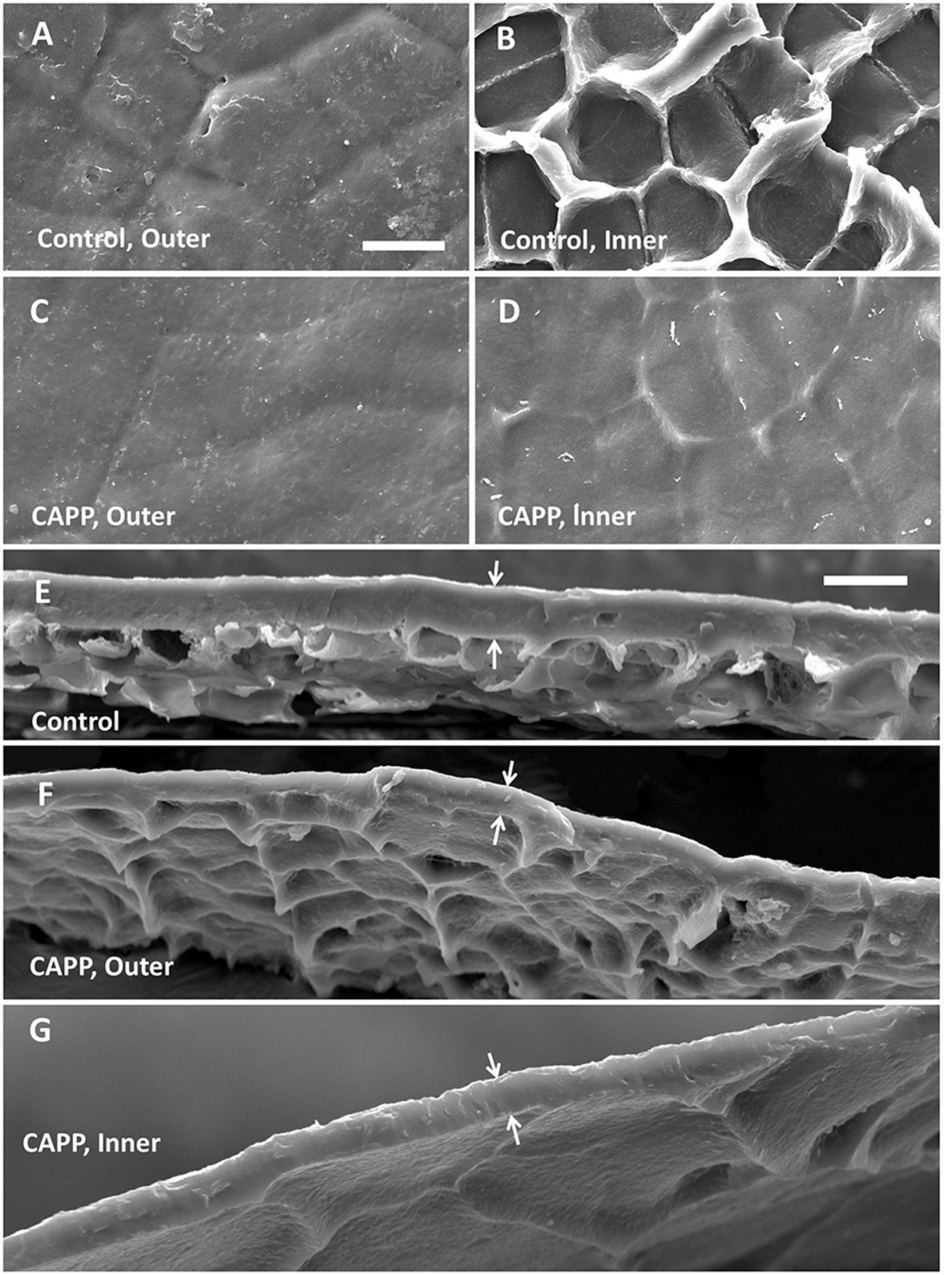
Scanning electron micrographs of outer surfaces **(A,C)**, inner surfaces **(B,D)**, and cross-sections **(E–G)** of cuticular membranes (CMs) of apple fruit (fed with ^13^C oleic acid at 69 days after full bloom [DAFB] and harvested at 178 DAFB) with ablation **(C,D,F,G)** and without ablation using a cold atmospheric pressure plasma (CAPP) **(A,B,E)** following wax extraction. The white arrows indicate the outer and inner surfaces of the CMs. The thickness of the CM differed significantly and was 11.0 ± 0.3 μm for the control, 7.4 ± 0.7 μm for the CAPP treatment of the outer side, and 5.5 ± 0.4 μm for the CAPP treatment of the inner side. Data on thickness represent means ± SE, *n* = 3–5.

Untreated CMs revealed a typical pattern of imprints of epidermal cell walls with slight depressions above the anticlinal cell walls when viewed from the outer surface (Figure 3A). On the inner surface, there were cuticular ridges above the anticlinal cell walls (Figure 3B). Exposure of outer or inner surfaces of CMs to CAPP resulted in significant ablations of the CM as indexed by significant decreases in CM thickness (Figures 3C–F). The cuticular ridges present on the inner surface of the CM above the anticlinal epidermal cell walls had almost disappeared after CAPP treatment for 20 min (Figures 3B,D,G).

**Figure 4:**
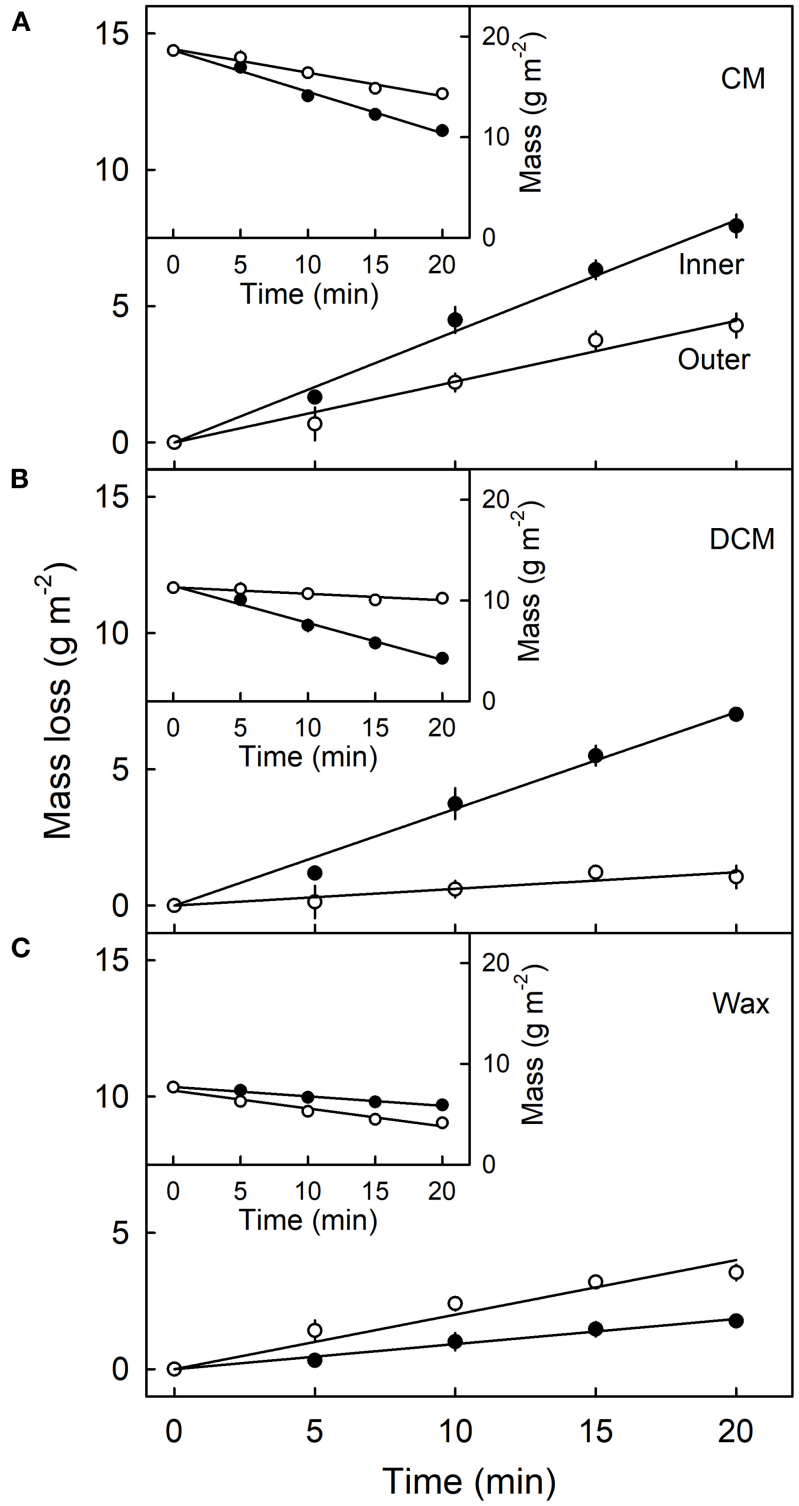
Effect of duration of ablation of the cuticular membrane (CM) of apple fruit using cold atmospheric pressure plasma (CAPP) on the mass loss (main figures) and absolute mass (inset figures) of the CM **(A)**, the dewaxed CM (DCM) **(B)** and the wax **(C)**. The inner or the outer surface of the CM was ablated using CAPP and the mass loss of the CM, the cutin and wax fraction determined. The fruit was fed 138 days after full bloom and harvested 14 days after the termination of the feeding. Data points represent means ± SE, *n* = 6–16.

Mass loss per unit area of the CM (Figure 4A), DCM (Figure 4B) and wax (Figure 4C) increased linearly as the duration of CAPP treatment of the CM increased. The mass loss of the CM and the DCM was lower when the morphological outer surface was ablated, as compared with the ablation of the morphological inner surface. The reverse applied for wax mass. Here, ablation of the outer surface induced a larger loss in wax mass as compared with ablation of the inner surface. This is not surprising considering the presence of epicuticular wax on the outer surface of the CM. Similar results were obtained with CM ablated after feeding at 103 or 138 DAFB (Table 1). When treating the outer surface, CAPP treatment ablated the entire epicuticular wax layer plus some amount of the cutin and cuticular wax, whereas CAPP treatment of the inner surface ablated cutin plus any cuticular wax only.

**Table 1:** Parameters of linear regression equations describing the relationships between mass loss (g m^−2^) and the duration of ablation using a cold atmospheric pressure plasma (CAPP) on the morphological inner and outer sides of isolated cuticular membranes (CM) of ‘Idared’ apple.

**Stage (DAFB)**	**Fraction**	**Morphological side**	**Slope ± SE**	**Coefficient of determination**
69	CM	Inner	0.33 ± 0.01	0.999***
		Outer	0.23 ± 0.02	0.956***
	DCM	Inner	0.36 ± 0.02	0.992***
		Outer	0.10 ± 0.01	0.932**
	Wax	Inner	0.08 ± 0.00	0.990***
		Outer	0.13 ± 0.02	0.916**
103	CM	Inner	0.41 ± 0.01	0.999***
		Outer	0.32 ± 0.01	0.994***
	DCM	Inner	0.32 ± 0.02	0.979***
		Outer	0.12 ± 0.01	0.983***
	Wax	Inner	0.04 ± 0.00	0.941**
		Outer	0.22 ± 0.02	0.975***
138	CM	Inner	0.42 ± 0.01	0.995***
		Outer	0.21 ± 0.01	0.986***
	DCM	Inner	0.33 ± 0.02	0.985***
		Outer	0.08 ± 0.01	0.949**
	Wax	Inner	0.10 ± 0.00	0.995***
		Outer	0.20 ± 0.01	0.983***

*The fruit was fed using ^13^C oleic acid for 7 d beginning at 69, 103, and 138 days after full bloom (DAFB). After 7 d, feeding was terminated. The fruit was harvested 14 d after terminating feeding and the CMs were isolated. Wax was extracted after CAPP treatment. Dewaxed CMs are referred to as DCMs. Since the intercept term was not significantly different from zero, all regression lines were forced through the origin*.

*SE standard error of the estimate*.

Significance of the coefficients of determination at the 1 and 0.1% levels are indicated by ^**^ and ^***^, respectively.

**Figure 5:**
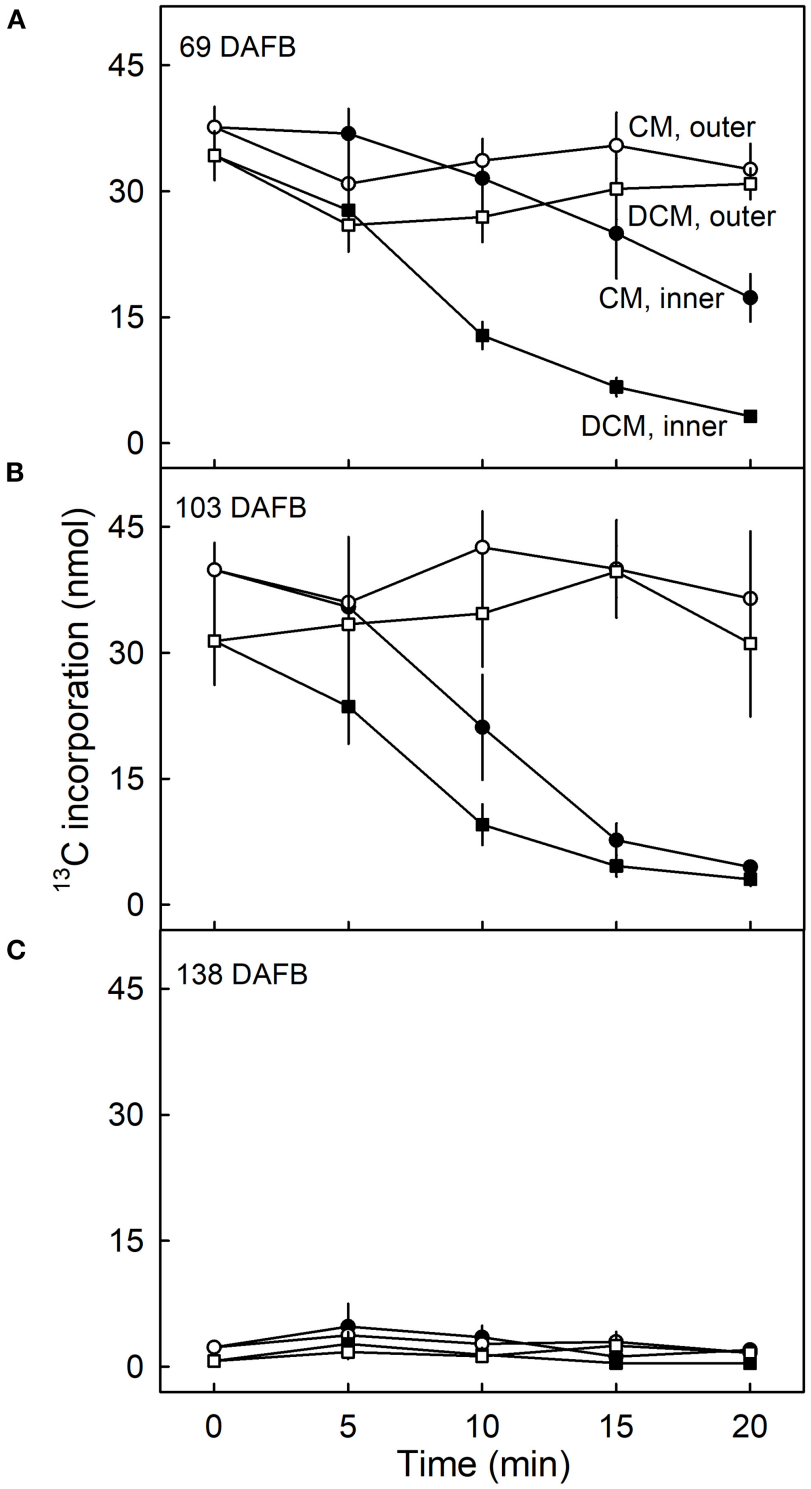
Effect of duration of ablation of cuticular membranes (CM) of developing apple fruit using cold atmospheric pressure plasma (CAPP) on the amount of ^13^C in the CM and the dewaxed CM (DCM). The inner or the outer surface of the CM was ablated using CAPP. Developing apple fruit were fed for 7 d using ^13^C labelled oleic acid at 69 **(A)**, 103 **(B)**, 138 **(C)** days after full bloom (DAFB). The fruit was sampled 14 d after the termination of the feeding and the CMs were isolated. Data points represent means ± SE, *n* = 6–8.

The amount of ^13^C in the CM and the DCM of fruit fed at 69 DAFB remained constant following ablation of the outer surface but decreased continuously when the inner surface was ablated (Figure 5A). Regardless of the duration of CAPP treatment of the inner side, the decrease in the amount of ^13^C in the DCM was always higher than that in the CM indicating that incorporation was in the cutin matrix and not or less in the wax.

Qualitatively and quantitatively similar results were obtained when analysing CMs and DCMs of fruit fed at 103 DAFB (Figure 5B). At 138 DAFB, the amounts of incorporation of ^13^C oleic acid in the CM and the DCM were low compared with those incorporated at 69 and 103 DAFB. There was no effect of CAPP treatment on ^13^C content at 138 DAFB (Figure 5C).

**Figure 6:**
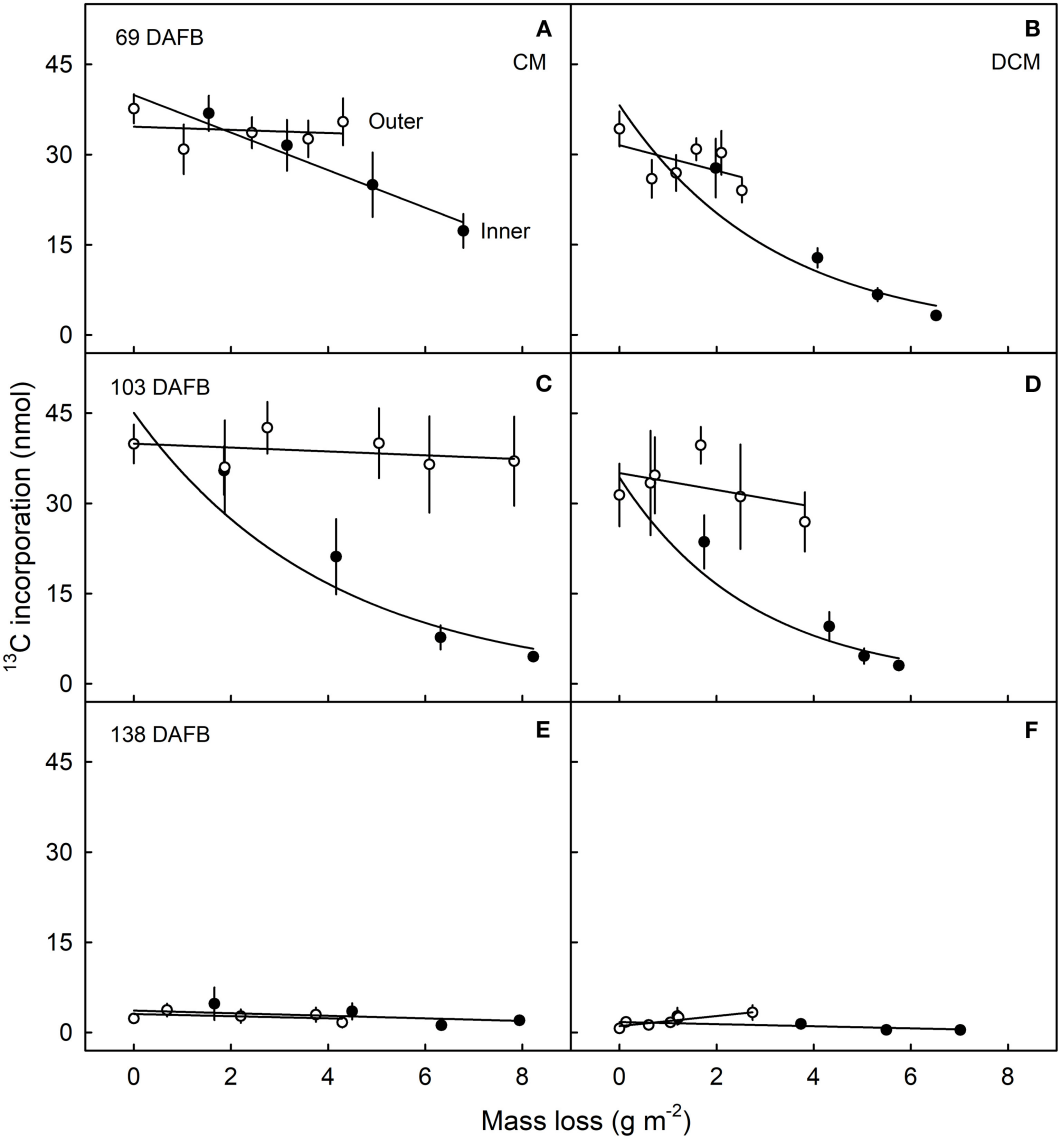
Relationship between the amount of ^13^C in the cuticular membrane (CM) **(A,C,E)** or in the dewaxed CM (DCM) **(B,D,F)** and the mass loss of the CM or DCM that resulted from ablation of the inner or outer surface of the CM using a cold atmospheric pressure plasma (CAPP). Developing apple fruit were fed for 7 d using ^13^C labelled oleic acid at 69 **(A,B)**, 103 **(C,D)**, and 138 **(E,F)** days after full bloom (DAFB). The fruit was sampled 14 d after the termination of feeding and the CMs were isolated. Data points represent means ± SE, *n* = 6–8. For data on the relationships between mass loss and duration of ablation of the CM by CAPP see Table 1.

Plotting the ^13^C content of the CM or the DCM as a function of the amount of mass loss following CAPP treatment revealed that increasing mass loss resulted in decreasing ^13^C content of CM and DCM when ablating their inner surfaces, but not when ablating their outer surfaces. With ablation of the outer surface of the CM, there was no relationship between the ^13^C amounts in the CM or DCM and the mass losses of the CM or DCM following ablation. Similar results were obtained for CMs and DCMs of fruit fed at 69 and 103 DAFB (Figures 6A–D). There were no effects of ablation on the ^13^C content of the CM or DCM for fruit fed at 138 DAFB (Figures 6E,F). At this stage of development, the deposition of CM has nearly ceased (Figures 2B,C).

**Figure 7:**
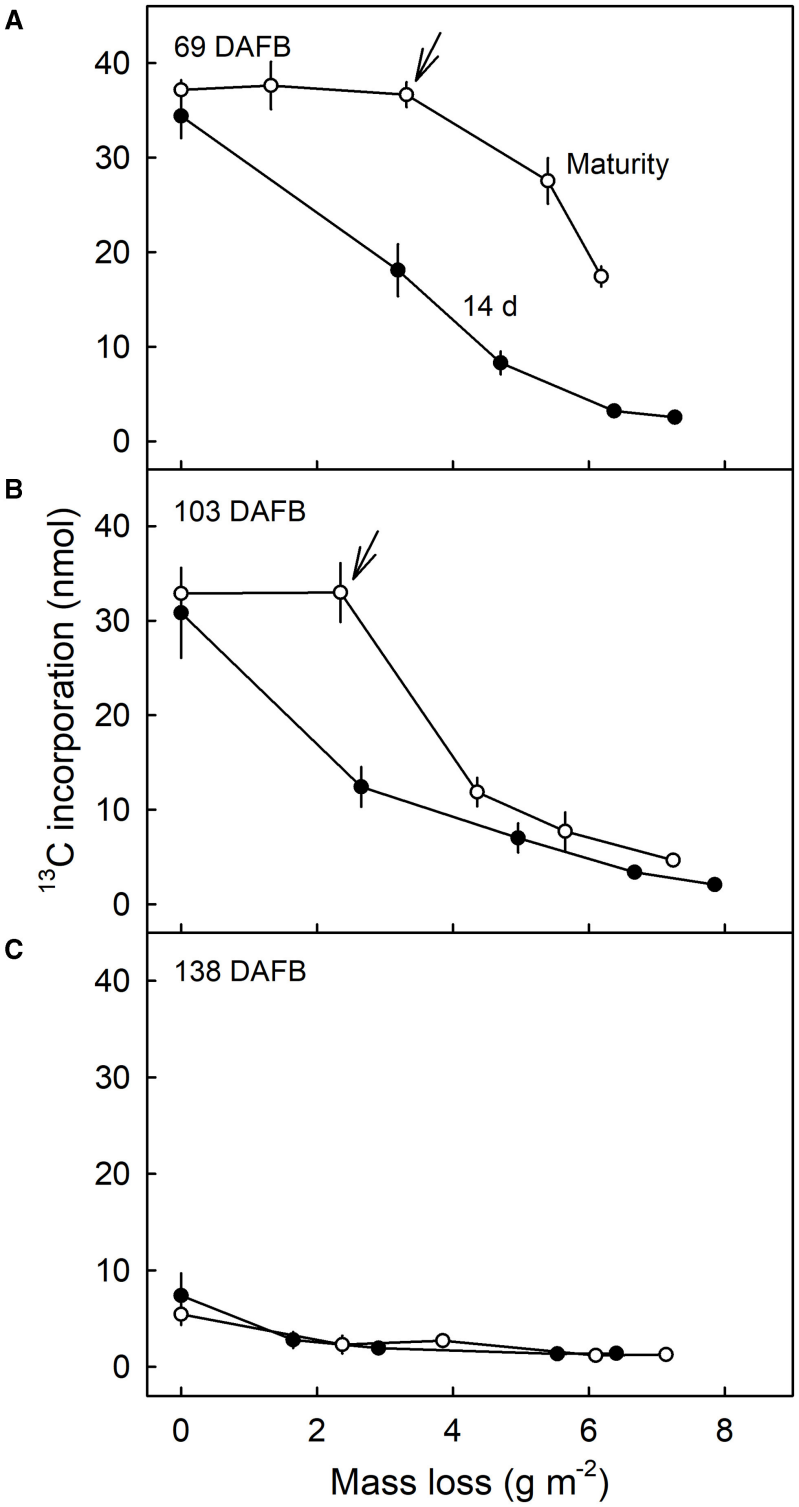
Relationship between the amount of ^13^C in the dewaxed cuticular membrane (DCM) and the mass loss of the DCM that resulted from ablation of the inner surface of the CM using a cold atmospheric pressure plasma (CAPP). Developing apple fruit were fed for 7 d using ^13^C labelled oleic acid at 69 **(A)**, 103 **(B)**, and 138 **(C)** days after full bloom (DAFB). The fruit was sampled either 14 d after the termination of the feeding or at maturity and the CMs isolated. Data points represent means ± SE, *n* = 8–10. For data on the relationships between mass loss and duration of ablation of the CM by CAPP see Supplementary Table 1.

Comparison of the ^13^C contents of the DCM of fruit harvested 14 d after feeding at 69 DAFB with those from fruit harvested at maturity revealed significant differences (Figure 7). CAPP ablation of the CM of the fruit 14 d after feeding yielded an immediate decrease in ^13^C content of the DCMs when carried out on the inner surface. However, when fruit was allowed to grow until maturity after feeding, ablation had no effects on the ^13^C content up to a mass loss of about 4 g m^−2^. Beyond this threshold, further ablation decreased the ^13^C content as mass loss increased (Figure 7A). For DCMs of fruit fed at 103 DAFB and harvested at maturity, the ^13^C contents began to decrease for a mass loss of about 2 g m^−2^ (Figure 7B). In DCMs of fruit harvested 14 d after the termination of feeding, the ^13^C content decreased continuously as mass loss increased. The fruit fed at 138 DAFB had very low ^13^C contents in the DCM. Consequently, ablation by CAPP had little effect on the ^13^C contents of the DCM (Figure 7C). This is consistent with the cessation of CM deposition at 138 DAFB (Figures 2B,C).

## Discussion

Our results evidence a radial gradient in the deposition and hence in the age of the cuticle of developing apple fruit. Feeding apple fruit with ^13^C oleic acid resulted in the incorporation and the deposition of labelled material on the inner surface of the CM. Consequently, the inner surface of the CM is younger, whereas the outer surface was deposited early on in fruit development, and so is older. The evidence for this conclusion is two-fold.

First, we obtained a gradient in ^13^C content of the DCM of fruit that (1) incorporated ^13^C oleic acid in the cuticle (69 and 103 DAFB) and that (2) was harvested 14 d after the termination of the feeding period. The rate of this decrease was initially rapid but slowed as the duration of CAPP treatment increased and as the mass-loss increased.

**Figure 8:**
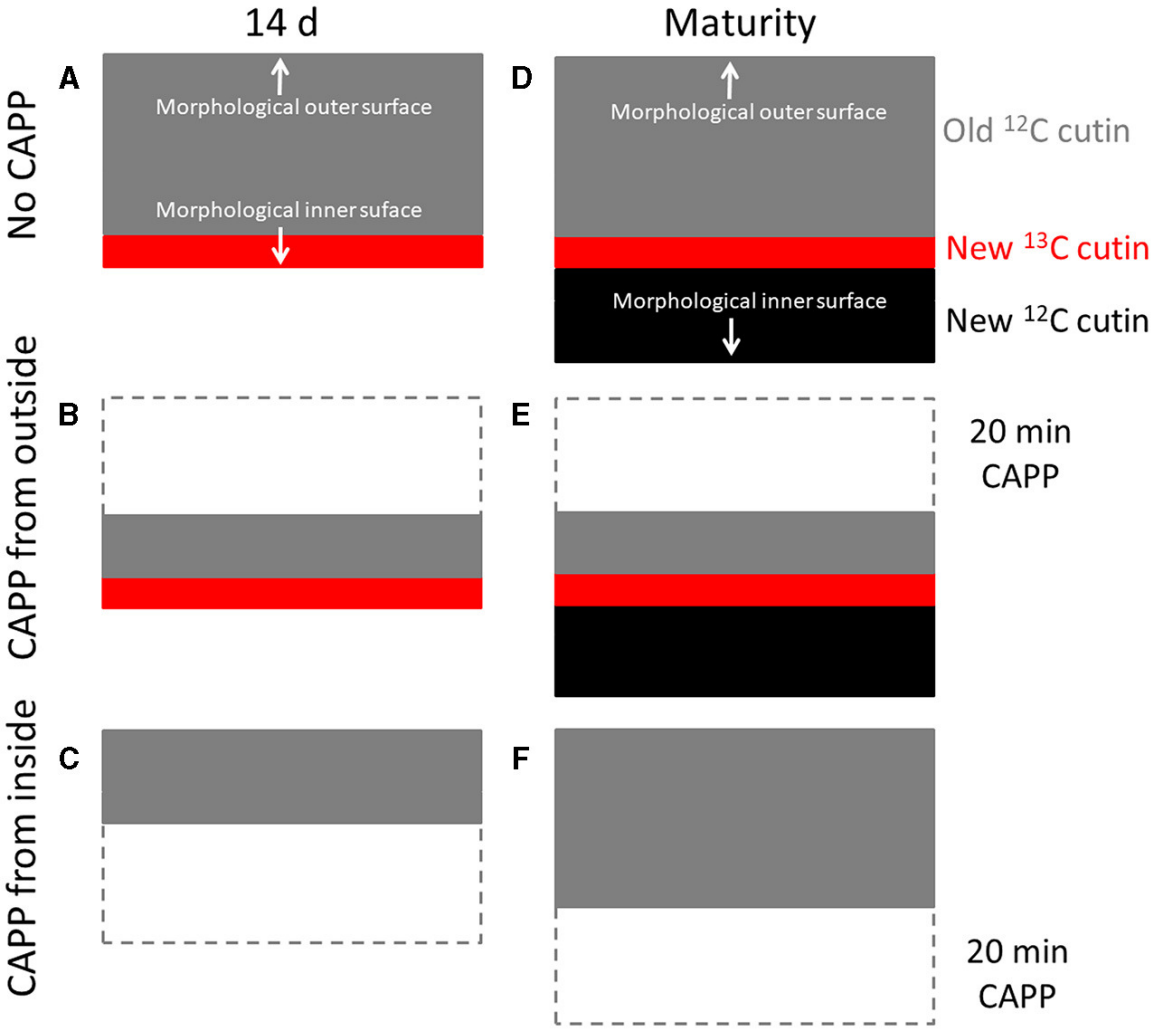
Sketch illustrating the location of the ^13^C labelled layer within the cuticular membrane (CM) that resulted from feeding the developing apple fruit using ^13^C labelled oleic acid for 7 d. The ^13^C labelled precursor is incorporated in the inner side of the CM **(A)**. Isolation of the CM at this stage and ablation from its outer side has no effect on the ^13^C content until the ^13^C labelled layer of the CM is ablated **(B)**, ablation from the inner side decreases the ^13^C content of the CM as mass loss increases **(C)**. In contrast, when fruit remains on the tree until maturity, CM deposition continues on the inner side. The layer resulting from feeding ^13^C oleic acid now “moves” into the CM **(D)**. Ablation from the outer side still has no effect on the ^13^C content until the ^13^C labelled layer of the CM is ablated **(E)**. When ablation from the inner side, ablation will not affect the ^13^C content of the CM until the labelled layer is reached **(F)**. Further ablation then will decrease the ^13^C content.

Second, when the fruit was fed with ^13^C oleic acid at 69 or 103 DAFB and then remained on the tree until maturity, the label was incorporated during the feeding period and immediately thereafter. However, un-labelled monomers were later incorporated in the cuticle on the inner surface. As cuticle deposition continued during development, the layer of the label was progressively overlaid and so “retreated” deeper into the cuticle as indicated in the sketch in Figure 8. Support for this view comes from the following observation. When the inner surface of the CM was ablated, short periods of ablation removed only the un-labelled portion of the cuticle, whereas longer ablations began to remove the labelled cuticle in ever deeper layers, and closer to the outer surface (Figure 8). The duration of the initial period without a decrease in ^13^C and the magnitude of the mass loss before the removal of the ^13^C labelled layer began depended on the thickness of the un-labelled layer, deposited after the termination of the feeding period. The duration of ablation before progressing into the labelled layer was longer for the feeding at 69 DAFB than for that at 103 DAFB. This interpretation is also consistent with the observation that treatment from the outer surface did not affect the ^13^C content of the polymer matrix. The above conclusions also account for the radial gradient in strain in apple fruit CM that has been reported previously (Khanal et al., 2014). Due to the earlier deposition, the outer CM has a longer history of strain and is, therefore, more strained, whereas the inner layer was deposited later and, hence, will have experienced less strain. This conclusion is also consistent with the structural characteristics of the apple cuticle (de Vries, 1968; Konarska, 2013). A cuticle proper (CP) that is rich in wax is distinguished from the underlying cuticular layer (CL) (Jeffree, 1996; Yeats and Rose, 2013). The CL was rich in cutin and contains embedded polysaccharides. The development of the CP precedes that of the CL (Jeffree, 2006).

The question arose as to what the chemical nature of the label might be incorporated in the cuticle. Since there was very little label associated with the wax, most of the incorporation was in the dewaxed CM. This indicates chemical binding, not simply partitioning into the CM (Si et al., 2021a). The two major constituents of the dewaxed CM are cutin and polysaccharides (Schreiber and Schönherr, 1990). Several arguments suggest this incorporation occurred in the cutin fraction.

First, the incorporation pattern was similar to that of CM deposition—i.e., it was higher during the early developmental stages, but lower in the late-stage close to maturity. This is consistent with the deposition pattern of cutin during the development of the apple fruit (Lai et al., 2016). Second, feeding ^14^C labelled oleic acids to apple skin discs resulted in the incorporation of the label in hydroxy C18 acids such as 18-hydroxyoctadecenoic acid, 10,18-dihydroxyoctadecanoic acid, and 9,10,18-trihydroxyoctadecanoic acid (Kolattukudy et al., 1971, 1973). These are major monomers of the apple fruit cutin (Walton and Kolattukudy, 1972; Straube et al., 2021). Also, until now the composition of apple cutin has been found to be consistent among all cultivars investigated (Holloway, 1973; Legay et al., 2017; Straube et al., 2021). Third, the cutin of apple fruit has been classified as a “mixed-type cutin” comprising C16 and C18 monomers (Holloway, 1982). This is in line with earlier observations from our laboratory, that when developing apple fruit were fed with ^14^C palmitic acid and ^14^C oleic acid incorporation in the CM of ^14^C palmitic acid was very much lower than of ^14^C oleic acid (Si et al., 2021a). This incorporation occurs in all apple cultivars investigated and at a rate that is significantly correlated with the mass of the CM per unit fruit surface area (Si et al., 2021b).

It may be argued that oleic acid is also a precursor for suberin and that the exposure of the apple fruit surface to the feeding solution may have resulted in microcracking and then periderm formation (Chen et al., 2020; Khanal et al., 2021). However, we considered this possibility extremely unlikely. First, ‘Idared’ is a cultivar that is known not to be susceptible to russeting (Khanal et al., 2013b). Russeting involves the formation of a periderm where the phellem typically has heavily suberized cell walls. Second, the feeding treatments in our study were done long after the period of greatest russet susceptibility was over (Chen et al., 2020; Khanal et al., 2021). Apple fruit is most susceptible to russet during the first 28 days after full bloom. Third, the apple fruit fed with oleic acid all had intact cuticles. There were no indications of either microcracking or russeting either to the naked eye or under SEM regardless of whether the fruit was harvested after a 7-day feeding plus the 14-d incorporation period or later at maturity. By this time, any russeting would have been visible on the fruit surface. Fourth, suberin is deposited inside the cell wall (Franke and Schreiber, 2007; Pollard et al., 2008). We obtained clean CMs after enzymatic isolation (see also Figure 3). Furthermore, a 5-min CAPP treatment of the inner surface decreased CM mass by about 8% (Khanal et al., 2014). This would have been sufficient to remove any hypothetical suberized cell walls that might have been present. This would have left no label in the dewaxed CM. However, in the fruit harvested at maturity the label from the early feeding was incorporated deep into the CM.

These arguments demonstrated that both, the label detected in the inner surface of the dewaxed CM from fruit harvested after the feeding and the incorporation period, and the label found deeper in the dewaxed CM of fruit harvested at maturity, most likely represent hydroxy C18 monomers polymerized in the cutin.

### Practical Implications

The deposition pattern of cutin on the inner side of the cuticle represents an important and critical mechanism that delays or prevents the formation of deep microcracks. A high rate of cutin deposition also maintains a minimum thickness of the CM during phases of rapid fruit expansion. Because the deposition occurs on the inner surface of the CM, the likelihood of the formation of microcracks that traverse the cuticle decreases. Microcracks that traverse the cuticle dramatically impair the barrier functions of the cuticle and trigger the formation of periderm, which led to russeting.

The results obtained in apple are thought likely to apply also to other fruit crop species that deposit cuticles throughout development. Further study should explore the possibility of stimulating the deposition of cutin during periods of rapid fruit growth, to help prevent deep propagation of microcracks.

## Data Availability Statement

The raw data supporting the conclusions of this article will be made available by the authors, without undue reservation.

## Author Contributions

BK and MK obtained the funds to support the study. YS, BK, and MK planned the experiments, wrote, revised, and edited the manuscript. YS and OS conducted the experiments. YS and BK analysed the data. All authors contributed to the article and approved the submitted version.

## Funding

This research was funded by a grant from the Deutsche Forschungsgemeinschaft (KH 374/2-1).

## Conflict of Interest

The authors declare that the research was conducted in the absence of any commercial or financial relationships that could be construed as a potential conflict of interest.

## Publisher's Note

All claims expressed in this article are solely those of the authors and do not necessarily represent those of their affiliated organizations, or those of the publisher, the editors and the reviewers. Any product that may be evaluated in this article, or claim that may be made by its manufacturer, is not guaranteed or endorsed by the publisher.
