# Supplementary material for: Direct Evidence for a Radial Gradient in Age of the Apple Fruit Cuticle
Source: Front Plant Sci. 2021 Oct 21;12:730837. doi: 10.3389/fpls.2021.730837 (PMC8567170; doi:10.3389/fpls.2021.730837)
Supplement: Supplementary file 1 [file Table_1.docx]

Supplementary Material

| **Supplementary Table 1.** Parameters of linear regression equations describing the relationships between the mass loss (g m^-2^) and duration of ablating the inner side of cuticular membrane (CM) of developing ‘Idared’ apple fruit using a cold atmospheric pressure plasma (CAPP). Fruit were fed for 7 d using a ^13^C oleic acid solution at 69, 103 and 138 days after full bloom (DAFB) and harvested 14 d after termination of the feeding period or at maturity. The CMs were isolated. Wax was extracted after CAPP treatment. Dewaxed CMs are referred to as DCMs.  Since the intercept term was not significantly different from zero, all regression lines were forced through the origin. | | | | |
| --- | --- | --- | --- | --- |
| Stage (DAFB) | Fraction | Harvest time | Slope ± SE | Coefficient of determination |
| 69 | CM | 14 d | 0.52 ± 0.03 | 0.982*** |
|  |  | Maturity | 0.44 ± 0.01 | 0.996*** |
|  | DCM | 14 d | 0.41 ± 0.03 | 0.980*** |
|  |  | Maturity | 0.35 ± 0.01 | 0.995*** |
|  | Wax | 14 d | 0.11 ± 0.01 | 0.953*** |
|  |  | Maturity | 0.08 ± 0.01 | 0.975*** |
| 103 | CM | 14 d | 0.54 ± 0.02 | 0.992*** |
|  |  | Maturity | 0.52 ± 0.03 | 0.989*** |
|  | DCM | 14 d | 0.43 ± 0.02 | 0.991*** |
|  |  | Maturity | 0.38 ± 0.01 | 0.994*** |
|  | Wax | 14 d | 0.12 ± 0.01 | 0.991*** |
|  |  | Maturity | 0.16 ± 0.02 | 0.956*** |
| 138 | CM | 14 d | 0.45 ± 0.01 | 0.996*** |
|  |  | Maturity | 0.53 ± 0.02 | 0.994*** |
|  | DCM | 14 d | 0.33 ± 0.01 | 0.993*** |
|  |  | Maturity | 0.38 ± 0.01 | 0.995*** |
|  | Wax | 14 d | 0.11 ± 0.01 | 0.956*** |
|  |  | Maturity | 0.16 ± 0.01 | 0.988*** |

*SE* standard error of the estimate

Significance of the coefficients of determination at the 0.1% level is indicated by ***.
